# Global trends of hand and wrist trauma: a systematic analysis of fracture and digit amputation using the Global Burden of Disease 2017 Study

**DOI:** 10.1136/injuryprev-2019-043495

**Published:** 2020-03-13

**Authors:** Christopher Stephen Crowe, Benjamin Ballard Massenburg, Shane Douglas Morrison, James Chang, Jeffrey Barton Friedrich, Gdiom Gebreheat Abady, Fares Alahdab, Vahid Alipour, Jalal Arabloo, Malke Asaad, Maciej Banach, Ali Bijani, Antonio Maria Borzì, Nikolay Ivanovich Briko, Chris D Castle, Daniel Youngwhan Cho, Michael T Chung, Ahmad Daryani, Gebre Teklemariam Demoz, Zachary V Dingels, Hoa Thi Do, Florian Fischer, Jack T Fox, Takeshi Fukumoto, Abadi Kahsu Gebre, Berhe Gebremichael, Juanita A Haagsma, Arvin Haj-Mirzaian, Demelash Woldeyohannes Handiso, Simon I Hay, Chi Linh Hoang, Seyed Sina Naghibi Irvani, Jacek Jerzy Jozwiak, Rohollah Kalhor, Amir Kasaeian, Yousef Saleh Khader, Rovshan Khalilov, Ejaz Ahmad Khan, Roba Khundkar, Sezer Kisa, Adnan Kisa, Zichen Liu, Marek Majdan, Navid Manafi, Ali Manafi, Ana-Laura Manda, Tuomo J Meretoja, Ted R Miller, Abdollah Mohammadian-Hafshejani, Reza Mohammadpourhodki, Mohammad A Mohseni Bandpei, Ali H Mokdad, Mukhammad David Naimzada, Duduzile Edith Ndwandwe, Cuong Tat Nguyen, Huong Lan Thi Nguyen, Andrew T Olagunju, Tinuke O Olagunju, Hai Quang Pham, Dimas Ria Angga Pribadi, Navid Rabiee, Kiana Ramezanzadeh, Kavitha Ranganathan, Nicholas L S Roberts, Leonardo Roever, Saeed Safari, Abdallah M Samy, Lidia Sanchez Riera, Saeed Shahabi, Catalin-Gabriel Smarandache, Dillon O Sylte, Berhe Etsay Tesfay, Bach Xuan Tran, Irfan Ullah, Parviz Vahedi, Amir Vahedian-Azimi, Theo Vos, Dawit Habte Woldeyes, Adam Belay Wondmieneh, Zhi-Jiang Zhang, Spencer L James

**Affiliations:** 1 Department of Surgery, Division of Plastic and Reconstructive Surgery, University of Washington, Seattle, Washington, USA; 2 Department of Surgery, Division of Plastic and Reconstructive Surgery, Stanford University, Palo Alto, California, USA; 3 College of Medicine and Health Sciences, Department of Nursing, Adigrat University, Adigrat, Ethiopia; 4 Evidence Based Practice Center, Mayo Clinic Foundation for Medical Education and Research, Rochester, Minnesota, USA; 5 Health Management and Economics Research Center, Iran University of Medical Sciences, Tehran, Iran; 6 Health Economics Department, Iran University of Medical Sciences, Tehran, Iran; 7 Plastic Surgery Department, University of Texas, Houston, Texas, USA; 8 Department of Hypertension, Medical University of Lodz, Lodz, Poland; 9 Polish Mothers’ Memorial Hospital Research Institute, Lodz, Poland; 10 Social Determinants of Health Research Center, Babol University of Medical Sciences, Babol, Iran; 11 Department of Clinical and Experimental Medicine, University of Catania, Catania, Italy; 12 Epidemiology and Evidence Based Medicine, I.M. Sechenov First Moscow State Medical University, Moscow, Russia; 13 Institute for Health Metrics and Evaluation, University of Washington, Seattle, Washington, USA; 14 Department of Otolaryngology - Head & Neck Surgery, Wayne State University, Detroit, Michigan, USA; 15 Toxoplasmosis Research Center, Mazandaran University of Medical Sciences, Sari, Iran; 16 School of Pharmacy, Aksum University, Aksum, Ethiopia; 17 Addis Ababa University, Addis Ababa, Ethiopia; 18 Center of Excellence in Public Health Nutrition, Nguyen Tat Thanh University, Ho Chi Minh City, Vietnam; 19 Department of Population Medicine and Health Services Research, Bielefeld University, Bielefeld, Germany; 20 Department of Dermatology, Kobe University, Kobe, Japan; 21 Gene Expression & Regulation Program, The Wistar Institute, Philadelphia, Pennsylvania, USA; 22 School of Pharmacy, Mekelle University, Mekelle, Ethiopia; 23 School of Public Health, Haramaya University, Harar, Ethiopia; 24 Department of Public Health, Erasmus University Medical Center, Rotterdam, Netherlands; 25 Department of Pharmacology, Tehran University of Medical Sciences, Tehran, Iran; 26 Obesity Research Center, Shahid Beheshti University of Medical Sciences, Tehran, Iran; 27 Public Health Department, Madda Walabu University, Bale Goba, Ethiopia; 28 Department of Health Metrics Sciences, School of Medicine, University of Washington, Seattle, Washington, USA; 29 Center of Excellence in Behavioral Medicine, Nguyen Tat Thanh University, Ho Chi Minh City, Vietnam; 30 Research Institute for Endocrine Sciences, Shahid Beheshti University of Medical Sciences, Tehran, Iran; 31 Department of Family Medicine and Public Health, University of Opole, Opole, Poland; 32 Social Determinants of Health Research Center, Research Institute for Prevention of Non-Communicable Diseases, Qazvin University of Medical Sciences, Qazvin, Iran; 33 Hematology-Oncology and Stem Cell Transplantation Research Center, Tehran University of Medical Sciences, Tehran, Iran; 34 Pars Advanced and Minimally Invasive Medical Manners Research Center, Iran University of Medical Sciences, Tehran, Iran; 35 Department of Public Health, Jordan University of Science and Technology, Irbid, Jordan; 36 Department of Physiology, Baku State University, Baku, Azerbaijan; 37 Epidemiology and Biostatistics Department, Health Services Academy, Islamabad, Pakistan; 38 Nuffield Department of Surgical Sciences, Oxford University Global Surgery Group, University of Oxford, Oxford, UK; 39 Department of Nursing and Health Promotion, Oslo Metropolitan University, Oslo, Norway; 40 School of Health Sciences, Kristiania University College, Oslo, Norway; 41 Department of Public Health, Trnava University, Trnava, Slovakia; 42 Ophthalmology Department, Iran University of Medical Sciences, Tehran, Iran; 43 Ophthalmology Department, University of Manitoba, Winnipeg, Manitoba, Canada; 44 Plastic Surgery Department, Iran University of Medical Sciences, Tehran, Iran; 45 Surgery Department, Emergency University Hospital Bucharest, Bucharest, Romania; 46 Breast Surgery Unit, Helsinki University Hospital, Helsinki, Finland; 47 University of Helsinki, Helsinki, Finland; 48 Pacific Institute for Research & Evaluation, Calverton, Maryland, USA; 49 School of Public Health, Curtin University, Perth, Western Australia, Australia; 50 Department of Epidemiology and Biostatistics, Shahrekord University of Medical Sciences, Shahrekord, Iran; 51 Department of Nursing, Shahroud University of Medical Sciences, Shahroud, Iran; 52 Pediatric Neurorehabilitation Research Center, University of Social Welfare and Rehabilitation Sciences, Tehran, Iran; 53 Laboratory of Public Health Indicators Analysis and Health Digitalization, Moscow Institute of Physics and Technology, Dolgoprudny, Russia; 54 Experimental Surgery and Oncology Laboratory, Kursk State Medical University of the Ministry of Health of the Russian Federation, Kursk, Russia; 55 Cochrane South Africa, South African Medical Research Council, Cape Town, South Africa; 56 Institute for Global Health Innovations, Duy Tan University, Hanoi, Vietnam; 57 Department of Psychiatry and Behavioural Neurosciences, McMaster University, Hamilton, Ontario, Canada; 58 Department of Psychiatry, University of Lagos, Lagos, Nigeria; 59 Department of Pathology and Molecular Medicine, McMaster University, Hamilton, Ontario, Canada; 60 Health Sciences Department, Muhammadiyah University of Surakarta, Sukoharjo, Indonesia; 61 Department of Chemistry, Sharif University of Technology, Tehran, Iran; 62 Department of Pharmacology, Shahid Beheshti University of Medical Sciences, Tehran, Iran; 63 Department of Surgery, University of Michigan, Ann Arbor, Michigan, USA; 64 Department of Clinical Research, Federal University of Uberlândia, Uberlândia, Brazil; 65 Emergency Department, Shahid Beheshti University of Medical Sciences, Tehran, Iran; 66 Department of Entomology, Ain Shams University, Cairo, Egypt; 67 Rheumatology Department, University Hospitals Bristol NHS Foundation Trust, Bristol, UK; 68 Institute of Bone and Joint Research, University of Sydney, Syndey, New South Wales, Australia; 69 Health Policy Research Center, Shiraz University of Medical Sciences, Shiraz, Iran; 70 Surgery 2nd Department - SUUB, Carol Davila University of Medicine and Pharmacy, Bucharest, Romania; 71 Surgery 2nd Department, Bucharest Emergency Hospital, Bucharest, Romania; 72 Department of Public Health, Adigrat University, Adigrat, Ethiopia; 73 Department of Health Economics, Hanoi Medical University, Hanoi, Vietnam; 74 Gomal Center of Biochemistry and Biotechnology, Gomal University, Dera Ismail Khan, Pakistan; 75 TB Culture Laboratory, Mufti Mehmood Memorial Teaching Hospital, Dera Ismail Khan, Pakistan; 76 Department of Anatomical Sciences, Maragheh University of Medical Sciences, Maragheh, Iran; 77 Trauma Research Center, Nursing Facility, Baqiyatallah University of Medical Sciences, Tehran, Iran; 78 Department of Human Anatomy, Histology, and Embryology, Bahir Dar University, Bahir Dar, Ethiopia; 79 Department of Nursing, Wollo University, Dessie, Ethiopia; 80 Department of Nursing and Midwifery, Addis Ababa University, Addis Ababa, Ethiopia; 81 Department of Preventive Medicine, Wuhan University, Wuhan, China

**Keywords:** burden of disease, hand injury, descriptive epidemiology

## Abstract

**Background:**

As global rates of mortality decrease, rates of non-fatal injury have increased, particularly in low Socio-demographic Index (SDI) nations. We hypothesised this global pattern of non-fatal injury would be demonstrated in regard to bony hand and wrist trauma over the 27-year study period.

**Methods:**

The Global Burden of Diseases, Injuries, and Risk Factors Study 2017 was used to estimate prevalence, age-standardised incidence and years lived with disability for hand trauma in 195 countries from 1990 to 2017. Individual injuries included hand and wrist fractures, thumb amputations and non-thumb digit amputations.

**Results:**

The global incidence of hand trauma has only modestly decreased since 1990. In 2017, the age-standardised incidence of hand and wrist fractures was 179 per 100 000 (95% uncertainty interval (UI) 146 to 217), whereas the less common injuries of thumb and non-thumb digit amputation were 24 (95% UI 17 to 34) and 56 (95% UI 43 to 74) per 100 000, respectively. Rates of injury vary greatly by region, and improvements have not been equally distributed. The highest burden of hand trauma is currently reported in high SDI countries. However, low-middle and middle SDI countries have increasing rates of hand trauma by as much at 25%.

**Conclusions:**

Certain regions are noted to have high rates of hand trauma over the study period. Low-middle and middle SDI countries, however, have demonstrated increasing rates of fracture and amputation over the last 27 years. This trend is concerning as access to quality and subspecialised surgical hand care is often limiting in these resource-limited regions.

## Introduction

As global rates of mortality decline, rates of non-fatal injury have increased, particularly in lower Socio-demographic Index (SDI) nations.^1^ Hand trauma occurs with considerable frequency,^2^ representing a significant proportion of non-fatal injuries requiring medical attention.^3^ Even seemingly minor hand and wrist injuries have the potential to result in chronic pain, lost productivity and decreased quality of life without proper management.^4^ Prompt and thorough evaluation by a hand specialist is often necessary to provide an optimal functional outcome, regardless of the injury pattern. Furthermore, rehabilitation of the injured hand is of great importance outside the acute period of injury.^5^ While expedient diagnosis, proper management (surgical or non-surgical) and long-term rehabilitation (eg, structured hand therapy to improve motion, strength, adaptive function, and so on) may be standard treatment protocol in high SDI regions, lower SDI countries likely do not have accessibility to such care.^6^

The Global Burden of Disease (GBD) study represents the most exhaustive estimation and review regarding trends of disease and injury worldwide.^7–10^ This study provides a comprehensive assessment of 354 diseases and injuries in 195 countries and territories from 1990 to 2017. Included in the current GBD analyses are estimates of prevalence, incidence, mortality, risk factors and disability-related health outcomes (eg, years lived with disability (YLD) and disability-adjusted life years). For non-fatal trauma, such as hand and wrist injuries, the GBD study has established a method for comparing these measures over time and by region. Estimates for non-fatal injury represent a new feature of the GBD study and were previously incorporated into measurements of disability.

Central to understanding the global burden of hand and wrist trauma is determining where these injuries and healthcare resources are most imbalanced.^11 12^ There has not yet been a systematic appraisal of the global burden of hand and wrist trauma for all countries, age groups and sexes. Current reviews of upper extremity injuries have instead focused on single institution or country-wide estimates, and thus are not generalisable to regions of different socioeconomic development.^13–16^ Given this relative paucity of data regarding the global pattern of these injuries, there is considerable value in estimating the burden of hand and wrist trauma. Since the burden of injury can be high in areas of the world that lack health data, there is also interest in estimating the incidence and prevalence of these conditions in all countries over a time period to provide information regarding the trends of hand injury. These estimates will likely influence future resource allocation and health system planning.

## Methods

Results from the Global Burden of Diseases, Injuries, and Risk Factors Study 2017 (GBD 2017) study were used; these are described in greater detail in the GBD summary publications^17–21^ (online supplementary appendix 1). The GBD 2017 results are publicly available via the GBD Results Tool (http://ghdx.healthdata.org/gbd-results-tool) and GBD Compare (https://vizhub.healthdata.org/gbd-compare/). All incorporated data sources meet minimum inclusion criteria as outlined by previously established guidelines. Notably, the GBD study complies with the Guidelines for Accurate and Transparent Health Estimates Reporting recommendations^22^ (online supplementary appendix 2). A brief description of the GBD study methods as they apply to this analysis is provided below.

10.1136/injuryprev-2019-043495.supp1 Supplementary data

10.1136/injuryprev-2019-043495.supp2 Supplementary data

First, in the hierarchy of causes and injuries, GBD 2017 differentiates injury cause from injury nature. The cause of injury designation includes causes such as road injury, falls, and fires, heat and hot substances. While cause of injury determines the cause of death in the event of fatality, the nature of injury that results from a cause determines the actual disability experienced in the event of a non-fatal injury. For example, if a fall occurs that leads to a hand fracture, the fall would be the cause of injury and the hand fracture would be the nature of injury. For GBD 2017, 30 mutually exclusive, collectively exhaustive causes of injuries were designated, with 47 natures of injury that could result from each cause.

In order to comprehensively measure the global burden of hand trauma, GBD 2017 first estimated the incidence of 30 different causes of injury. This list includes road injuries and their subtypes; falls; fires, heat and hot substances; interpersonal violence; self-harm; and others. GBD 2017 uses a wide array of incidence data for each cause of injury, which are documented and catalogued in detail in GBD literature and in the Global Health Data Exchange (http://ghdx.healthdata.org). Incidence data included literature studies, survey data, surveillance data, outpatient (clinic) data, hospital data and insurance claims data. Each data source used was extracted, processed, reviewed and analysed as part of the GBD 2017 study. Once data for each cause of injury were available, GBD 2017 modelled the incidence of each injury cause using DisMod-MR V.2.1—a Bayesian meta-regression tool that uses a compartmental model framework to reconcile incidence, cause-specific mortality and remission.^23^ Further details regarding the modelling approach for each cause of injury are available in previous GBD publications.^1^

After each cause of injury was modelled, the incidence of each cause was split into incidence of each cause–nature combination. This process is based on clinical data where both cause and nature of injury were coded. The clinical sources and analytical method used for this process are described in more detail elsewhere.^24^ In this manner, GBD 2017 measured the proportion of each cause of injury that would lead to a hand or wrist fracture, thumb amputation or finger amputation when it was the most disabling injury sustained in a given case. Each nature of injury is assigned to a GBD disability weight to measure YLDs. Finally, the rates are summed across causes such that the overall incidence, prevalence and YLDs for each nature of injury (including hand and wrist fractures, thumb amputations and finger amputations) can be computed. The final results from this process were obtained, reported and described for this research study.

Socio-demographic Index (SDI) is a marker of development status used by the GBD study and for this analysis. In brief, it is calculated using the total fertility rate under the age of 25, mean education for those aged 15 and older and lag distributed income per capita. An SDI of 0 and 1 would reflect a minimum and maximum level of development relevant to health, respectively.

Similar to other GBD analyses,^1 7–9^ uncertainty is measured at various steps of the analytical process using the sample size, SE or original uncertainty interval (UI). Uncertainty is maintained in a distribution of 1000 draws and is then propagated in draw space through each analytical step. The 95% UIs reported in this study are the 25th and 975th values of the ordered 1000 values across draws.

The analytical processes were conducted in Python V.2.7, Stata V.13.1 or R V.3.3. The statistical code used in steps of this analytical process is available online (http://www.ghdx.healthdata.org). Results with additional detail by age, sex, year and location can be downloaded at ghdx.healthdata.org.

## Results

Global rates of bony hand trauma have decreased slightly over the last 27 years (table 1). In 2017, an estimated 178.9 (95% UI 145.8 to 216.8) age-standardised hand and wrist fractures per 100 000 individuals occurred worldwide, representing a 2.6% decrease from 1990. Furthermore, there were 24.1 thumb (95% UI 17.4 to 33.9) and 56.0 (95% UI 43.4 to 74.0) non-thumb digit amputations per 100 000. Males comprise the majority of those who sustain hand and wrist fractures (1.8:1 male-to-female incidence ratio), thumb amputations (1.9:1 incidence ratio) and non-thumb digit amputations (2.3:1 incidence ratio). Males, however, have experienced a greater reduction in the incidence of these injuries since 1990 compared with females.

**Table 1:** Global incidence of hand and wrist trauma and digit amputations

	Incident cases in 2017 (95% UI), n × 100 000			Age-standardised incidence rate (ASIR) in 2017 per 100 000, estimate (95% UI)			Estimated percentage change in ASIR, 1990–2017		
	Wrist and hand fractures	Thumb amputation	Non-thumb amputation	Wrist and hand fractures	Thumb amputation	Non-thumb amputation	Wrist and hand fractures	Thumb amputation	Non-thumb amputation
Overall	137.5 (111.6 to 166.8)	18.6 (13.3 to 26.1)	43.0 (33.3 to 56.7)	178.9 (145.8 to 216.8)	24.1 (17.4 to 33.9)	56.0 (43.4 to 74.0)	−2.6	−4.4	−3.7
Sex									
Male	87.4 (72.7 to 104.5)	12.1 (8.8 to 16.6)	29.8 (23.3 to 38.7)	227.0 (118.4 to 271.8)	31.4 (22.9 to 43.2)	77.3 (60.6 to 100.2)	−3.2	−4.6	−4.3
Female	50.0 (38.9 to 64.0)	6.5 (4.4 to 9.7)	13.2 (9.8 to 18.3)	129.7 (100.9 to 165.5)	16.8 (11.5 to 25.2)	34.5 (25.5 to 48.3)	−0.9	−2.3	−2.0
Socio-demographic Index									
Low	19.6 (16.2 to 23.7)	2.3 (1.6 to 3.1)	6.8 (5.3 to 9.0)	160.6 (132.3 to 193.6)	18.6 (13.4 to 25.6)	55.3 (42.7 to 73.1)	−4.4	2.7	−20.1
Low-middle	25.5 (21.1 to 30.6)	3.2 (2.3 to 4.3)	8.7 (6.8 to 11.4)	152.8 (125.7 to 183.1)	19.0 (13.9 to 25.8)	51.7 (40.1 to 67.3)	15.9	14.7	16.4
Middle	24.4 (20.3 to 29.5)	3.9 (2.9 to 5.4)	7.8 (6.0 to 10.1)	115.2 (96.1 to 140.0)	18.5 (13.5 to 25.4)	36.8 (28.4 to 48.3)	25.5	19.7	22.5
High-middle	34.0 (27.4 to 41.6)	4.3 (3.0 to 6.1)	10.2 (7.8 to 13.5)	243.9 (194.7 to 299.5)	30.4 (21.1 to 44.4)	72.8 (55.2 to 97.3)	−1.4	−1.4	−4.0
High	33.6 (26.5 to 41.6)	4.9 (3.4 to 7.2)	9.4 (6.9 to 12.7)	297.8 (237.2 to 366.0)	44.0 (30.2 to 64.7)	85.1 (63.0 to 114.5)	−9.1	−12.1	−8.7
Region									
High-income Asia Pacific	4.8 (3.7 to 6.0)	0.9 (0.7 to 1.4)	1.4 (1.0 to 1.9)	275.2 (216.7 to 343.7)	53.6 (36.9 to 79.2)	81.6 (59.7 to 109.0)	4.3	7.3	7.5
Central Asia	3.1 (2.5 to 3.9)	0.3 (0.2 to 0.5)	0.9 (0.7 to 1.3)	342.1 (270.2 to 429.9)	36.7 (24.6 to 56.9)	99.9 (74.0 to 138.7)	−1.7	−0.9	−0.9
East Asia	20.1 (16.4 to 24.6)	0.3 (0.3 to 0.4)	6.0 (4.5 to 7.9)	131.1 (107.9 to 159.0)	20.6 (14.7 to 28.8)	38.5 (29.2 to 50.5)	63.0	46.5	56.9
South Asia	25.9 (21.2 to 31.1)	0.4 (0.3 to 0.5)	7.9 (6.0 to 10.4)	149.0 (121.6 to 180.1)	20.5 (14.8 to 27.9)	44.9 (34.5 to 59.7)	14.9	13.8	7.9
Southeast Asia	4.7 (4.0 to 5.7)	0.8 (0.6 to 1.0)	1.7 (1.3 to 2.2)	71.2 (59.8 to 84.9)	11.6 (8.6 to 15.6)	25.2 (19.5 to 32.8)	10.7	19.1	−4.3
Australasia	1.8 (1.4 to 2.3)	0.2 (0.1 to 0.3)	0.6 (0.4 to 0.8)	652.6 (506.7 to 819.9)	74.2 (48.7 to 112.2)	207.5 (149.4 to 286.0)	13.7	13.3	14.6
Oceania	0.2 (0.1 to 0.2)	0.02 (0.01 to (0.03)	0.1 (0.0 to 0.1)	122.0 (102.7 to 144.5)	15.4 (11.2 to 20.7)	41.6 (32.0 to 53.2)	36.7	34.2	21.1
Central sub-Saharan Africa	1.7 (1.4 to 2.1)	0.2 (0.1 to 0.3)	0.6 (0.5 to 0.8)	149.6 (125.4 to 180.2)	17.5 (12.6 to 24.2)	54.6 (41.5 to 72.5)	−3.2	3.0	−7.0
Eastern sub-Saharan Africa	6.5 (5.3 to 7.9)	0.8 (0.6 to 1.2)	2.3 (1.8 to 3.1)	177.0 (144.4 to 216.7)	23.3 (16.7 to 33.1)	61.7 (46.9 to 82.2)	−29.9	−1.4	−64.9
North Africa and Middle East	13.2 (10.7 to 17.2)	1.3 (1.0 to 1.9)	5.7 (3.9 to 9.2)	216.5 (175.1 to 282.3)	21.9 (15.9 to 30.6)	93.4 (63.5 to 149.0)	6.0	−21.9	43.3
Southern sub-Saharan Africa	1.2 (1.0 to 1.4)	0.1 (0.1 to 0.2)	0.4 (0.3 to 0.5)	152.0 (125.1 to 181.2)	18.5 (13.4 to 25.3)	50.8 (38.8 to 66.2)	−6.6	−6.2	−7.6
Western sub-Saharan Africa	5.7 (4.7 to 6.9)	0.7 (0.5 to 0.9)	2.0 (1.5 to 2.6)	141.9 (116.7 to 171.2)	16.7 (12.0 to 23.1)	48.6 (38.1 to 64.1)	−3.3	0.4	−6.2
Central Europe	7.6 (5.8 to 10.0)	0.7 (0.5 to 1.3)	2.1 (1.5 to 3.0)	666.8 (511.2 to 865.3)	68.8 (43.5 to 112.8)	154.1 (113.4 to 215.2)	−0.9	0.6	−3.5
Eastern Europe	11.1 (8.7 to 14.2)	1.2 (0.8 to 1.8)	3.2 (2.3 to 4.4)	544.7 (427.6 to 691.2)	57.3 (37.9 to 90.2)	184.3 (131.6 to 260.4)	−1.9	−3.8	0.6
Western Europe	13.4 (10.5 to 16.8)	1.4 (0.9 to 2.1)	3.7 (2.7 to 5.0)	318.8 (249.9 to 396.3)	33.6 (22.4 to 52.4)	89.2 (65.7 to 121.9)	−1.5	−0.4	−2.1
Andean Latin America	0.8 (0.6 to 0.9)	0.08 (0.06 to 0.1)	0.2 (0.2 to 0.3)	127.4 (102.3 to 156.8)	13.8 (9.4 to 20.4)	38.4 (28.9 to 51.6)	−1.8	4.5	−36.1
Central Latin America	2.7 (2.1 to 3.3)	0.5 (0.3 to 0.6)	0.8 (0.6 to 1.1)	106.1 (84.5 to 130.7)	18.0 (12.5 to 25.8)	31.4 (23.6 to 41.2)	−2.7	−2.6	−14.8
Southern Latin America	2.1 (1.7 to 2.5)	0.2 (0.2 to 0.3)	0.4 (0.3 to 0.5)	317.1 (253.6 to 387.9)	35.6 (24.5 to 52.0)	102.2 (76.0 to 138.4)	12.4	12.2	15.9
Tropical Latin America	2.3 (1.8 to 3.0)	0.5 (0.4 to 0.8)	0.6 (0.4 to 0.8)	104.5 (81.8 to 135.3)	23.5 (16.5 to 34.1)	26.7 (19.9 to 35.5)	27.7	23.6	23.4
High-income North America	7.8 (6.2 to 9.9)	1.7 (1.1 to 2.5)	2.2 (1.6 to 3.0)	210.7 (167.4 to 262.8)	46.5 (32.0 to 67.7)	60.0 (43.5 to 81.1)	−31.4	−35	−35.7
Caribbean	0.6 (0.5 to 0.8)	0.09 (0.06 to 0.1)	0.2 (0.2 to 0.3)	137.8 (110.8 to 168.3)	19.7 (12.3 to 29.1)	46.1 (35.1 to 60.8)	34.3	76.8	46.0

UI, uncertainty interval.

**Figure 1:**
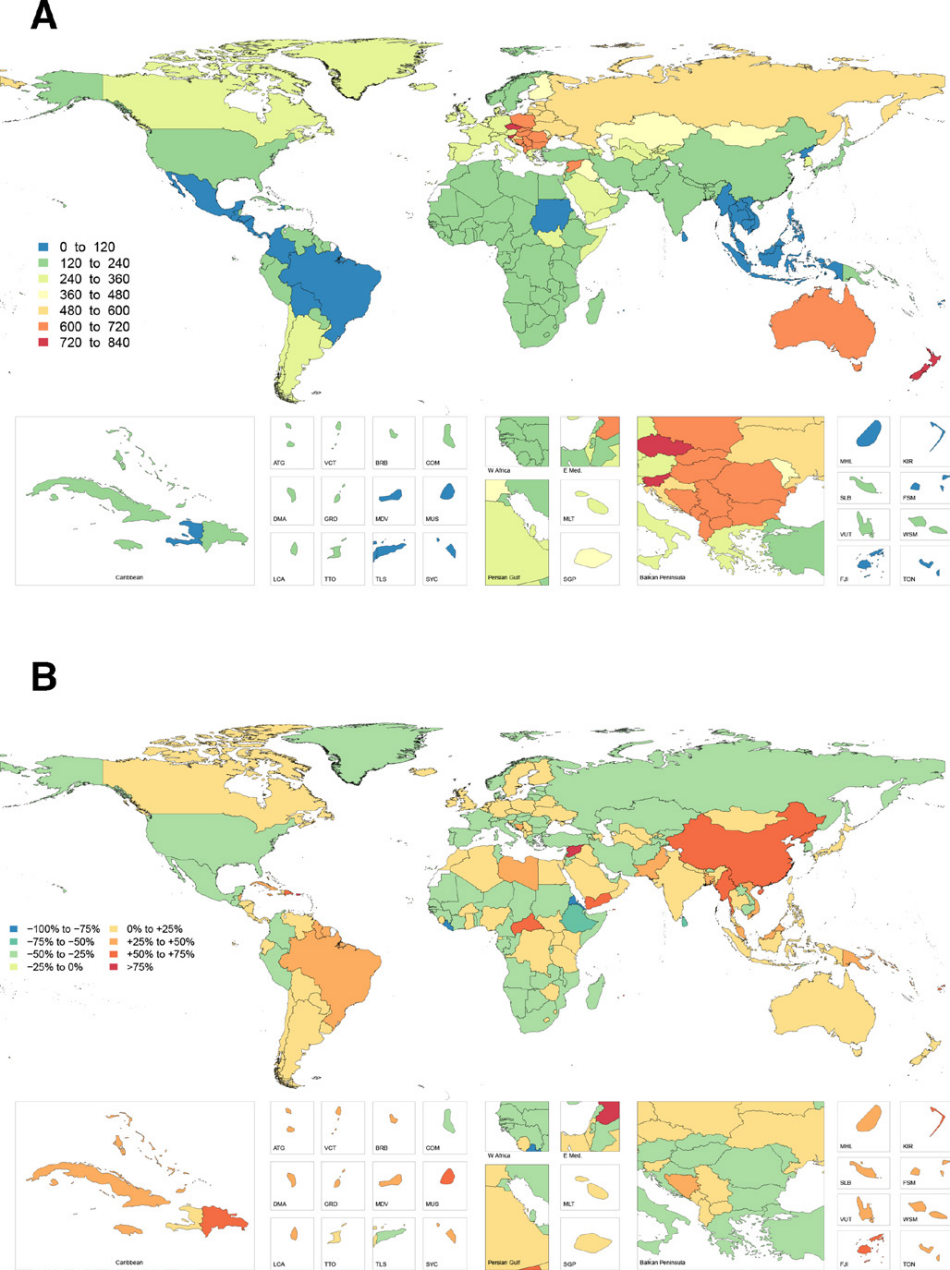
Age-standardised incidence rate of hand and wrist fractures in 2017 (A) and percentage change in hand and wrist fracture incidence rate from 1990 to 2017 (B).

The highest overall number of hand and wrist fractures in 2017 was observed in South and East Asia; however, the age-standardised rate of this injury was highest in Central Europe, Australasia and Eastern Europe, corresponding to 666.8 (95% UI 511.2 to 865.3), 652.6 (95% UI 506.7 to 819.9) and 544.7 (95% UI 427.6 to 691.2) injuries per 100 000, respectively (figure 1). Within these regions, New Zealand, Czech Republic, Slovenia, Slovakia, Poland and Australia had the highest age-standardised rates of hand and wrist fractures. Incidence was lowest in Southeast Asia and Tropical and Central Latin America, corresponding to 71.2 (95% UI 59.8 to 84.9), 104.5 (95% UI 81.8 to 135.3) and 106.1 (95% UI 84.5 to 131.1) injuries per 100 000, respectively. The countries Timor-Leste, Laos, Mauritius, Indonesia and Philippines had the lowest incidence overall (online supplementary table 1).

10.1136/injuryprev-2019-043495.supp3 Supplementary data

**Figure 2:**
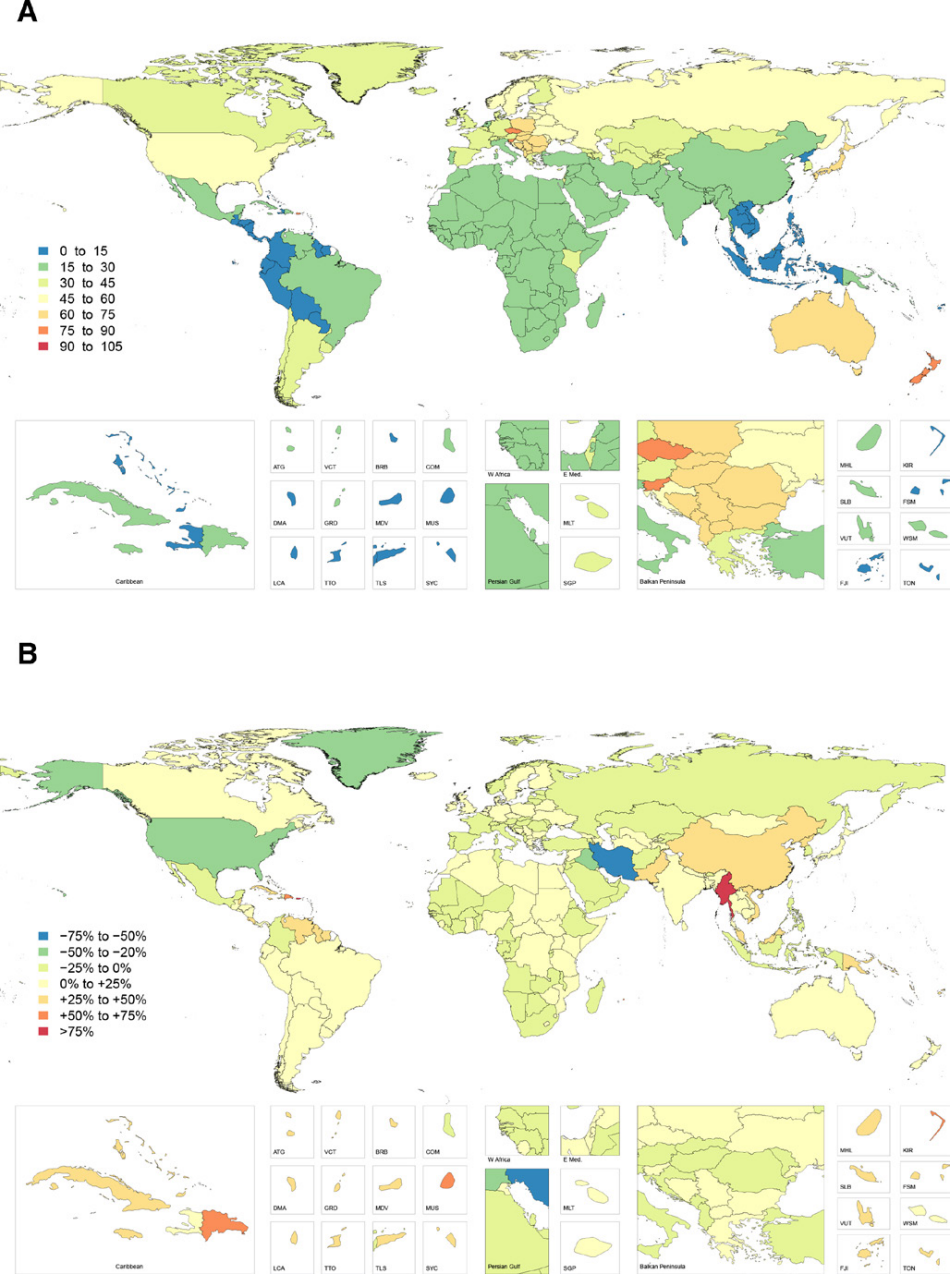
Age-standardised incidence rate of thumb amputations in 2017 (A) and percentage change in thumb amputation incidence rate from 1990 to 2017 (B).

**Figure 3:**
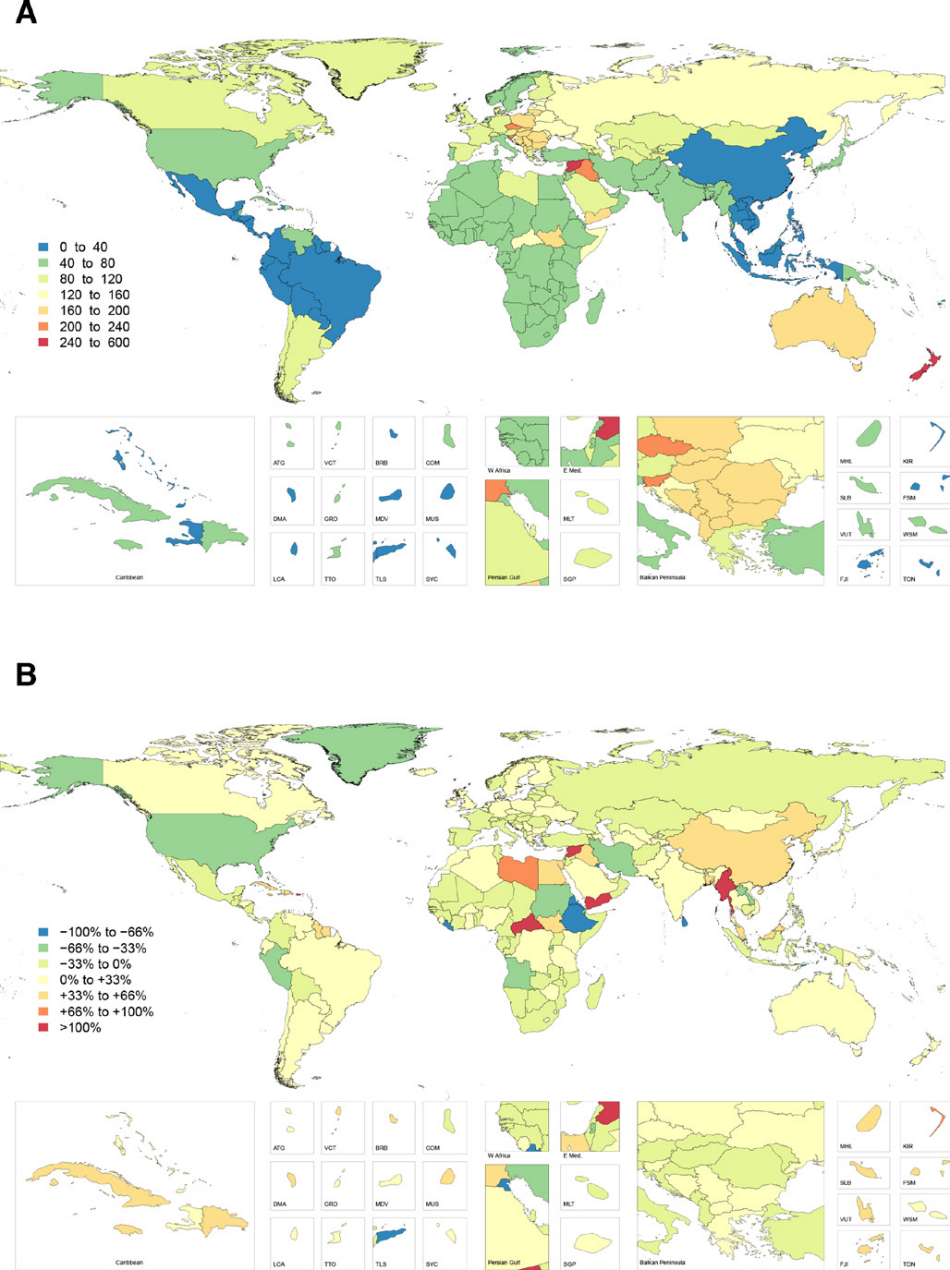
Age-standardised incidence rate of non-thumb digit amputations in 2017 (A) and percentage change in non-thumb digit amputation incidence rate from 1990 to 2017 (B).

The highest overall number of digit amputations was reported in high-income North America and Western and Eastern Europe. The highest incidence of thumb amputation was again observed in Australasia, followed by Central and Eastern Europe, corresponding to 74.2 (95% UI 48.7 to 112.2), 68.8 (95% UI 43.5 to 112.8) and 57.3 (95% UI 37.9 to 90.2) injuries per 100 000, respectively (figure 2). The highest incidence of non-thumb digit amputation was observed in Australasia, followed by Eastern and Central Europe. This corresponded to 207.5 (95% UI 149.4 to 286.0), 184.3 (95% UI 131.6 to 260.4) and 154.1 (95% UI 113.4 to 215.2) injuries per 100 000, respectively (figure 3). The incidence of digit amputations (thumb and non-thumb) was lowest in Oceania, Andean Latin America and the Caribbean. The countries Timor-Leste, Laos, Philippines and Mauritius had the lowest incidence of thumb amputation, while Indonesia, Timor-Leste, Laos and Mauritius had the lowest incidence of non-thumb amputation.

The most significant increase in injury incidence by region since 1990 was noted in East Asia—with a 63%, 47% and 57% increase in the age-standardised rate of fracture, thumb amputation and non-thumb digit amputation, respectively. China and North Korea make up the majority of these increases. Other regions with increases (eg, Oceania, Caribbean, Tropical and Southern Latin America) were substantial, but not to the same magnitude as East Asia. Variable patterns of change were seen in sub-Saharan Africa and the Middle East. High-income North America, however, experienced a substantial reduction in the rates of fracture, thumb amputation and non-thumb digit amputation.

**Figure 4:**
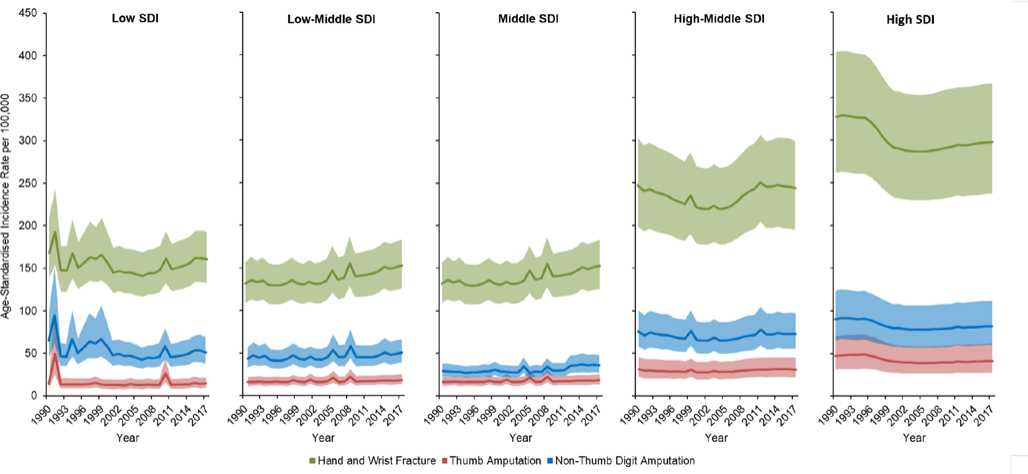
Age-standardised incidence of hand and wrist trauma by Socio-demographic Index (SDI).

Over the study period, high SDI countries had the highest reported age-standardised incidence of hand and wrist fracture, thumb amputation and non-thumb digit amputation, corresponding to 297.8 (95% UI 237.2 to 366.0), 44.0 (95% UI 30.2 to 64.7) and 85.1 (95% UI 63.0 to 114.5) injuries per 100 000, respectively (figure 4). Middle SDI countries had the lowest age-standardised incidence of hand and wrist trauma, corresponding to 115.2 (95% UI 96.1 to 140.0) hand and wrist fractures, 18.5 (95% UI 13.5 to 25.4) thumb amputations and 36.8 (95% UI 28.4 to 48.3) non-thumb digit amputations per 100 000, respectively.

Paralleling the reduction in high-income North America, the high SDI country group experienced a substantial decrease in the rates of these injuries over the last 27 years, with an estimated 9% decrease in hand and wrist fractures, 12% decrease in thumb amputation and 9% decrease in non-thumb digit amputation. During the same period, however, hand and wrist fractures increased by 16% and 26%, thumb amputations by 15% and 20% and non-thumb digit amputations by 16% and 23% in low-middle and middle SDI groups, respectively.

**Figure 5:**
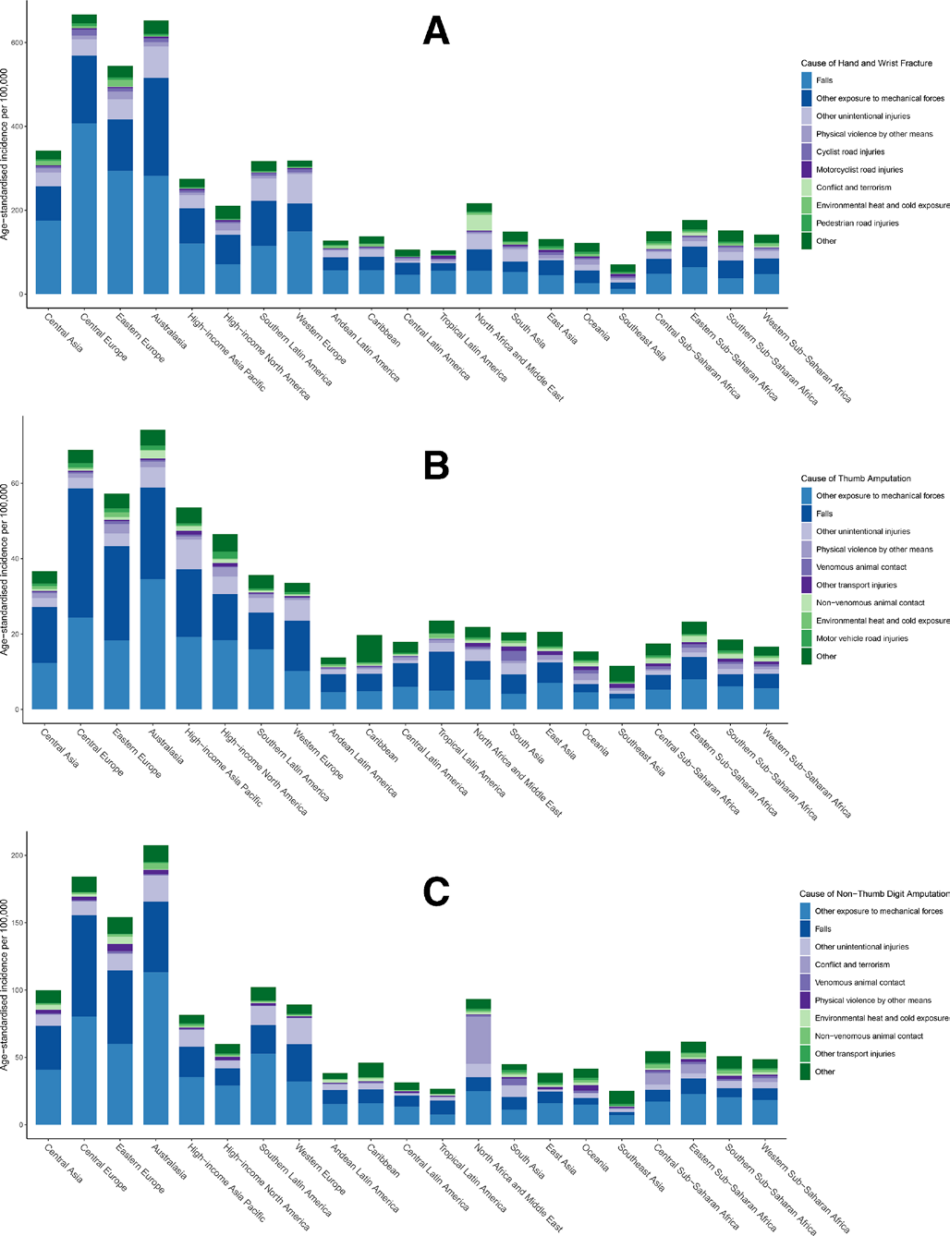
Causes of hand and wrist fracture (A), thumb amputation (B) and non-thumb digit amputation (C) by region.

The greatest proportion of hand and wrist fractures occurred secondary to falls, followed by other exposures to mechanical forces and unintentional injuries (figure 5). Causes of hand and wrist fracture are overall similar in Central and Eastern Europe and Australasia. The greatest proportion of digit amputations is due to exposures to otherwise unspecified mechanical forces, which likely represents industrial injuries. Notably, conflict and terrorism account for a greater number of all bony hand injuries in North Africa and the Middle East.

Trends of disability, specifically YLDs, parallel trends of the incidence of hand trauma (table 2). Thumb amputation accounts for the greatest burden of disability globally at 10.5 (5.0–19.7) per 100 000, as compared with hand and wrist fracture at 4.1 (2.1–7.3) and non-thumb digit amputation at 9.4 (3.5–19.9) YLDs per 100 000, respectively. As before, the highest rate of YLDs was observed in Australasia and Central and Eastern Europe. The high SDI group had the greatest observed burden of disability related to hand trauma overall; however, the low-middle and middle SDI groups experienced increases in YLDs over the last 27 years, paralleling the rising reported incidence in these regions.

**Table 2:** Global disability of hand and wrist trauma and digit amputations

	Rate of age-standardised years lived with disability (ASYLD) in 2017 (95% UI) per 100 000			Estimated percentage change in rate of ASYLDs (1990–2017)		
	Wrist and hand fractures	Thumb amputation	Non-thumb amputation	Wrist and hand fractures	Thumb amputation	Non-thumb amputation
Overall	4.1 (2.1 to 7.3)	10.5 (5.0 to 19.7)	9.4 (3.5 to 19.9)	−4.7	−5.7	−3.1
Sex						
Male	5.1 (2.6 to 9.1)	13.7 (6.6 to 26.0)	13.0 (4.8 to 27.6)	−2.5	−6.2	−3.0
Female	3.1 (1.6 to 5.5)	7.8 (3.5 to 13.6)	5.9 (2.2 to 12.5)	−4.7	−6.1	−3.3
Socio-demographic Index						
Low	3.3 (1.8 to 5.7)	7.4 (3.6 to 13.9)	9.1 (3.3 to 19.6)	7.1	9.2	9.3
Low-middle	3.1 (1.7 to 5.5)	7.3 (3.5 to 13.8)	7.9 (2.9 to 17.2)	15.2	15.5	14.9
Middle	2.7 (1.4 to 4.9)	7.5 (3.6 to 14.0)	5.6 (2.1 to 11.9)	24.2	23.6	24.4
High-middle	5.2 (2.7 to 9.5)	12.6 (6.0 to 23.7)	12.0 (4.4 to 25.5)	−6.0	0.1	−4.4
High	6.2 (3.1 to 11.2)	18.3 (8.8 to 34.1)	14.3 (5.3 to 30.2)	−9.4	−12.6	−7.7
Region						
High-income Asia Pacific	6.2 (3.1 to 11.2)	23.4 (11.1 to 43.4	14.1 (5.2 to 29.6)	5.6	8.8	13.1
Central Asia	7.0 (3.6 to 12.8)	15.2 (7.2 to 28.7)	16.7 (6.2 to 35.2)	−1.9	1.0	3.9
East Asia	3.2 (1.7 to 5.8)	8.6 (4.1 to 16.4)	6.5 (2.4 to 14.0)	55.4	49.9	53.8
South Asia	3.0 (1.6 to 5.4)	8.1 (3.9 to 15.3)	7.2 (2.6 to 15.1)	20.3	20.8	14.3
Southeast Asia	2.0 (1.1 to 3.5)	4.5 (2.2 to 8.3)	4.2 (1.6 to 9.1)	19.1	19.4	24.3
Australasia	12.0 (5.8 to 21.5)	32.3 (15.3 to 59.9)	35.6 (13.3 to 74.9)	11.8	14.3	13.7
Oceania	2.9 (1.5 to 5.1)	6.6 (3.2 to 12.2)	7.3 (2.7 to 15.5)	47.0	43.1	46.9
Central sub-Saharan Africa	3.5 (1.9 to 5.9)	6.3 (3.1 to 12.0)	9.5 (3.4 to 21.4)	4.0	0.5	18.9
Eastern sub-Saharan Africa	4.3 (2.4 to 7.1)	8.8 (4.2 to 16.6)	11.0 (4.0 to 24.5)	−2.2	0.2	2.3
North Africa and Middle East	4.2 (2.4 to 7.2)	9.0 (4.4 to 17.0)	13.0 (4.7 to 29.0)	−4.0	2.4	3.7
Southern sub-Saharan Africa	3.2 (1.7 to 5.8)	7.4 (3.6 to 14.1)	8.3 (3.0 to 17.5)	−11.1	−6.7	−7.8
Western sub-Saharan Africa	2.9 (1.5 to 5.2)	6.3 (3.0 to 11.8)	7.3 (2.7 to 15.4)	−1.7	−1.5	3.1
Central Europe	12.7 (6.4 to 23.1)	28.0 (13.5 to 51.9)	24.8 (9.2 to 52.0)	−1.9	2.5	−1.4
Eastern Europe	10.6 (5.3 to 19.2)	23.4 (11.1 to 43.4)	29.8 (11.1 to 62.2)	−2.6	−0.9	5.0
Western Europe	5.9 (3.0 to 10.8)	14.4 (6.8 to 26.4)	15.2 (5.6 to 32.2)	−1.8	0.3	0.9
Andean Latin America	2.6 (1.4 to 4.7)	5.6 (2.7 to 10.5)	6.8 (2.5 to 14.5)	5.6	11.3	7.9
Central Latin America	2.4 (1.3 to 4.2)	7.1 (3.4 to 13.3)	5.5 (2.0 to 11.8)	−3.7	−0.9	−5.8
Southern Latin America	6.0 (3.0 to 10.9)	14.6 (7.0 to 27.4)	16.7 (6.2 to 35.3)	7.3	13.5	15.0
Tropical Latin America	2.6 (1.4 to 4.7)	8.9 (4.3 to 16.9)	4.1 (1.5 to 8.7)	30.9	25.4	22.6
High-income North America	5.0 (2.5 to 9.1)	18.3 (8.8 to 34.3)	9.7 (3.6 to 20.9)	−32.4	−40	−37.4
Caribbean	3.1 (1.7 to 5.2)	9.2 (4.2 to 18.3)	8.1 (2.9 to 17.7)	47.6	116.3	61.2

UI, uncertainty interval.

## Discussion

Hand and wrist injury has the potential to result in significant impairment, affecting both social and vocational activities.^4^ Unfortunately, these injuries are overwhelmingly common, affecting all ages, sexes and geographic regions. Prior descriptions of the epidemiology of hand and wrist injuries have focused on isolated data sets, and have not compared the frequency and effect of these injuries by geography and income group worldwide. This is the first study that aims to measure the global incidence and resultant disability of hand and wrist fracture, thumb amputation and non-thumb digit amputation using data collected between 1990 and 2017 as part of the GBD 2017 study.

Although hand trauma is frequent and affects all demographic groups, these injuries are not equally distributed and significant variation by region and SDI group exists. The first discernible pattern is that fractures and amputations appear to be most concentrated in high-middle and high SDI groups. This trend is consistent with other forms of non-fatal trauma as estimated in the GBD 2017 study,^25^ but may be related to the more comprehensive reporting of injuries. In particular, Australasia and Central and Eastern Europe were observed to have the highest incidence of hand and wrist trauma per capita, while East and South Asia had the highest total number of hand and wrist injuries overall. The rates of fracture and digit amputation in sub-Saharan Africa and the Middle East were lower than expected, and are likely greater than estimated secondary to a paucity of medical data collection and poor access to medical care.

The inequitable distribution of fatal and non-fatal traumatic injuries has been well studied in Europe.^26–28^ While some European countries fall into the high SDI group and have similar rates of trauma relative to high-income Asian and North American countries, others are relatively low income. Research has suggested that additional sources of trauma in Europe are secondary to military conflict, which has taken place every year prior to and during the 27-year study period.^29^ Other studies have demonstrated a strong association between alcohol use and mortality, specifically in Central and Eastern Europe.^30 31^ Furthermore, there has also been a significant influx of migrant populations, with approximately 76 million international immigrants residing in Europe in 2015^32^ and many of those individuals migrating from conflict zones.^33^ This may skew the rate of injuries documented in Central and Eastern Europe.^34^

Although the burden of traumatic injuries is documented in Australia and New Zealand,^35^ the specific reason for why rates of hand trauma are observed to be comparatively high in the Australasian region remains unclear. One study comparing injury patterns between the USA and New Zealand found that rates of fatal and severe non-fatal injuries were higher in New Zealand.^36^ Explanations for this disparity included higher populations in rural environments, road design, differences in trauma system implementation and differences in legislation and public policy.

The second pattern noted in this study is that bony hand and wrist trauma appears to be increasing at a significant rate in the low-middle and middle SDI groups, whereas it is observed to be decreasing in the higher SDI groups. This phenomenon may be due to a number of different causes. As access to healthcare improves, more individuals may seek medical care than previously, and thus an increasing number of injuries are recorded as a result. Another explanation is that industrial job availability may predispose individuals to occupational hand and wrist trauma that may have not been possible previously. Similarly, the so-called ‘motorization’ of lower SDI countries may also affect rates of trauma. For instance, the growing proportion of individuals operating personal vehicles in low-income countries^37^ combined with poor traffic safety standards likely results in more collisions.^38^ Another cause may be the improved survivability of traumatic injuries in these countries stemming from implementation of health safety measures and development of trauma systems. As trauma victims survive what may have otherwise been fatal injuries, increasing rates of non-fatal polytrauma may be noted. Lastly, medical record keeping may also be improving in these regions, now reflecting an estimate closer to the true number of hand injuries.

The third pattern of hand injury is the distribution of hand injury by sex. Females represented approximately one-third of all hand injuries captured in this study. Global reductions in age-standardised incidence over the 27-year period were approximately 50% of their male counterparts. When divided by age group, male injuries demonstrated a bimodal distribution across all SDI groups with one peak occurring between 15 and 40 years of age, and another peak after 80 years of age.^25^ We speculate that the first group captures occupational hand injuries and also corresponds to increased rates of trauma in general for younger men.^39^ This peak becomes more pronounced from 1990 to 2017 for low-middle and middle SDI countries, but is reduced in the high SDI group over that time period. For females, this first peak does not exist no matter the SDI group or year. Although speculative, this may relate to the different occupational hazards to which men and women are exposed. Males may demonstrate a greater reduction because males tend to make up a majority of hand injuries in the workplace.^40 41^

Regardless of the underlying cause, bony injuries of the hand follow universal principles of fracture management—establish anatomic reduction, maintain immobilisation for adequate bony healing and subsequently mobilise the joints to prevent stiffness.^42 43^ The technologies available to achieve reduction and immobilisation will vary widely based on the medical resources and capabilities of the country or region in question. Lower SDI countries will not have access to proper diagnostic equipment and a trained surgical workforce, thus placing the populations they serve at greater risk of experiencing the long-term sequelae of these injuries. Estimates of surgical workforce and unmet surgical need, as expected, show that low-income countries have the greatest unmet need.^44^ Based on this analysis, 93% of the people living in sub-Saharan Africa and 97% of those in South Asia are without access to safe and affordable surgical care, as compared with 3.6% of the population in higher income regions. This undoubtedly signals an imbalance in the rate of hand injuries and the specific resources needed to adequately diagnose and treat those injuries.

Despite the lesser frequency of digit amputations as compared with fracture, loss of a finger, and more notably a thumb, has the potential to result in more impairment and subsequent disability. Fractures can go on to heal with little permanent deficit or deformity, depending on the degree of comminution and displacement of fracture pieces. Loss of a digit, however, will often result in at least some degree of permanent functional deficit (eg, range of motion, fine pinch, power grasp, strength, sensibility).^45^ This is well illustrated by the higher disability weight and years lived with disability (YLD) for thumb amputations globally. Optimal management after a digit amputation considerably depends on the mechanism of injury and level of amputation. Even in high-resource areas, a digit replantation may not be ideal, and thus ensuring good postinjury hand function relies on a well-planned revision amputation. Secondary reconstructive modalities (eg, osteoplastic lengthening, free tissue transfer, ray resection) following a digit amputation require a high level of expertise. Furthermore, access to specialised occupational and physical therapists, as well as prosthetists, may not be possible in lower income regions.

Improving the burden of hand injuries, like all disease states, depends both on prevention of the injury and reduction of associated impairment when it occurs. One study of risk factors of work-related acute hand injuries in the USA found that malfunctioning or incorrectly used equipment, in addition to subjective factors such as being distracted or rushed, contributed to occupational hand trauma.^46^ Policy mandating the implantation of industrial and occupational safety protocols—including the use of protective equipment—is critical for reducing the number of workplace incidents. Similarly, the introduction of safety devices for heavy machinery and occupational equipment would help prevent high-energy and mangling hand injuries, which can have devastating consequences for patients.^47^ Targeted safety campaigns and education regarding the use of protective vocational and recreational gear could also be promoted at a number of levels.^48^ The primary impediment for these efforts is the cost associated with their implementation, which may be prohibitive in lower SDI countries.

After an injury occurs, expedient diagnosis and proper management mitigates the long-term sequelae of bony hand trauma. Primary care providers, who will see the majority of bony hand injuries, should receive adequate education regarding management of hand and wrist fractures. Thus, outreach missions should focus on providing clinical services for those in need, and educating local practitioners—particularly non-specialists. In addition, emergency response systems should also be designed in such a way that hospitals with specialised hand and microsurgical services are prioritised in instances of digit amputation. Uncertainty regarding where a patient should be taken initially may cause untoward delays in care. Lastly, the impact of trained physical and occupational therapists following injury should never be underestimated. Global outreach should prioritise acute management of the fracture, and focus on the aftercare and rehabilitation of these injuries.

There are several limitations inherent in this study. First, this analysis only allows estimation for bony injuries of the hand, which represent a subset of the total hand trauma incurred globally. Soft tissue injuries, including acute burns, skin laceration, tendon laceration, vascular injury and nerve damage, will not be captured in an isolated manner. Additionally, hand burn contractures also make up a substantial burden of hand disability and are not estimated in this study.^49–51^ The sequelae of burn injury, especially in the upper extremity, require the expertise of a reconstructive surgeon to treat. These soft tissue injuries make up a substantial proportion of the overall burden of hand trauma, and thus actual rates of hand and wrist trauma will be far greater when all types of trauma are considered.

Furthermore, estimates rely on the availability of clinical and hospital data, which may be lacking in lower income regions and conflict zones. In these circumstances, estimation models rely more heavily on covariates. The current GBD method for estimating the cause–nature relationships of hand and wrist injuries (ie, hand and wrist fractures and digital amputations) depends on dual-coded hospital data, which are not always available in every country.

Next, due to data constraints in GBD 2017, disability weights may not accurately reflect specific outcomes for specific types of injury. For instance, a non-displaced fracture of the fifth metacarpal will not experience the same degree of disability compared with someone who sustains a comminuted distal radius fracture, and these outcomes may vary even within a specific patient population receiving care at the same facility. This level of detail is difficult to capture at the population level. As previously noted, the current GBD study design uses an injury severity hierarchy model, which is determined by the most severe nature of injury sustained for a given cause. Therefore, the model will ignore certain injuries in instances of polytrauma. For example, in circumstances in which an individual sustains a hand injury in conjunction with a more serious injury such as an intracranial bleed, the hand injury will not be accounted for. Lastly, a considerable limitation of the current investigation is that assumptions regarding the exact cause and mechanism of injury were made as a result of restrictions within the available data set. This limitation will be addressed with further iterations of the GBD study.

## Conclusion

High rates of bony hand and wrist injuries are noted in Central Europe, Eastern Europe and Australasia, which are consistent with patterns found in other anatomic zones of injury (eg, facial fracture, traumatic brain injury and spinal cord injury).^38^ In low-middle and middle SDI countries, increasing rates of fracture and amputation are observed over the 27-year study period. Patients in these countries are less likely to have access to quality and subspecialised surgical hand care.

The comparative reporting of hand and wrist injuries by region and SDI will allow for design and implementation of preventative measures and effective management strategies. We anticipate that future GBD studies will provide more granular data to gauge the effectiveness of these efforts over time.

**What is already known on the subject**

- Fractures of the hand and wrist, as well as digit amputations, are debilitating injuries that occur in all regions and income groups, and occur by a variety of mechanisms.
- Bony hand trauma occurs globally with high incidence; however, the resulting impairment and disability depends on the severity of injury, prompt diagnosis and proper treatment.

**What this study adds**

- In 2017, there were approximately 18 million hand and wrist fractures, 2 million thumb amputations and 4 million non-thumb digit amputations worldwide.
- Bony hand injuries, including digit amputation, occur in greatest number in South and East Asia, but appear to be most concentrated (per capita) in Central Europe, Eastern Europe and Australasia.
- Whereas the rate of these hand injuries is decreasing in the higher Socio-demographic Index (SDI) countries, low-middle and middle SDI regions have experienced an increasing rate of hand injuries over the past 27 years.
